# Supplementary material for: Factors Affecting the Number of Pollen Grains per Male Strobilus in Japanese Cedar (Cryptomeria japonica)
Source: Plants (Basel). 2021 Apr 23;10(5):856. doi: 10.3390/plants10050856 (PMC8146487; doi:10.3390/plants10050856)
Supplement: Supplementary file 1 [file plants-10-00856-s001.zip › Supplementary20210414/supple figure.pdf]

plants

Supplementary Figure 1

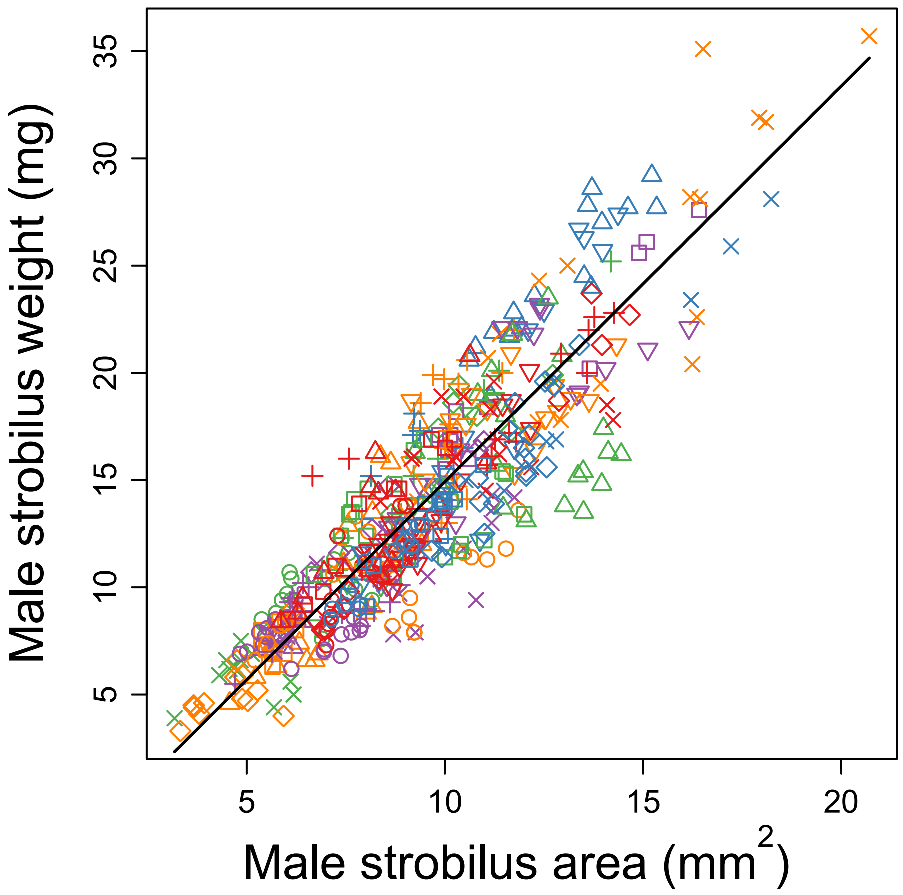

Scheme 1. Scatterplot of male strobilus weight and area. All samples are shown in this figure. Weight and area are strongly correlated (r = 0.892). Different clones are represented by different symbols (see Table 1).
